# Physical Activity Among Predominantly White Middle-Aged and Older US Adults During the SARS-CoV-2 Pandemic: Results From a National Longitudinal Survey

**DOI:** 10.3389/fpubh.2021.652197

**Published:** 2021-04-13

**Authors:** Rodney P. Joseph, Keenan A. Pituch, M. Aaron Guest, Molly Maxfield, Allie Peckham, David W. Coon, Wonsun Kim, Shelby L. Langer

**Affiliations:** ^1^Center for Health Promotion and Disease Prevention, Edson College of Nursing and Health Innovation, Arizona State University, Phoenix, AZ, United States; ^2^Edson College of Nursing and Health Innovation, Arizona State University, Phoenix, AZ, United States; ^3^Center for Innovation in Healthy and Resilient Aging, Edson College of Nursing and Health Innovation, Arizona State University, Phoenix, AZ, United States

**Keywords:** COVID-19, exercise, physical activity, older adults, United States

## Abstract

**Background:** The first COVID-19 case in the US was diagnosed late January 2020. In the subsequent months, cases grew exponentially. By March 2020, SARS-CoV-2 (the novel coronavirus that causes COVID-19) was a global pandemic and the US declared a national emergency. To mitigate transmission, federal guidelines were established for social and physical distancing. These events disrupted daily routines of individuals around the world, including Americans. The impact of the pandemic on PA patterns of Americans is largely unknown, especially among those at greater risk for severe COVID-19 outcomes. The aim of this study was to assess levels of PA over time during the pandemic among US adults aged >50 years.

**Methods:** Data were collected as part of a web-based, longitudinal, 3-wave study examining health and well-being among adults aged > 50. PA data were collected at Waves 2 and 3 using the International Physical Activity Questionnaire-Short Form (IPAQ-SF). At Wave 2 (conducted mid-May to early June, 2020), participants completed the IPAQ-SF twice, once in reference to a typical 7-day period before the pandemic, and again in reference to the past 7 days. At Wave 3 (conducted mid-June to early July 2020), participants completed the IPAQ-SF once, with reference to the past 7 days. Potential predictors of PA change were collected using items from previously established surveys and included demographic characteristics, pre-pandemic PA levels, perceived COVID-19 threat, self-rated general health, and number of chronic disease conditions.

**Results:** Respondents (*N* = 589) had a mean age of 63 ± 7.39 years and were mostly female (88%) and non-Hispanic White (96%). Mean MET-min/week across the three time-referents were 2,904 (pre-pandemic), 1,682 (Wave 2 past 7-days), and 2,001 (Wave 3 past 7-days), with PA declining between the first and second time referents (*d* = −0.45, *p* < 0.001) and remaining below pre-pandemic levels at the third (*d* = −0.34, *p* < 0.001). Changes over time were predicted by pre-pandemic PA and self-rated general health (*p's* < .05).

**Conclusions:** Effective strategies are needed to promote safe and socially-distanced PA among adults aged >50 years until the risk of contracting COVID-19 subsides. In the post-pandemic era, PA programming will be imperative to address pandemic-associated declines in PA.

## Introduction

The first known COVID-19 case in the United States (US) was diagnosed January 20, 2020 (1). Over the proceeding weeks, the number of cases grew exponentially and a national emergency was declared on March 13, 2020 (2). In an effort to reduce transmission of SARS-CoV-2 (the novel coronavirus that causes COVID-19), mitigation strategies were widely implemented in the US. These strategies included national guidelines for physical and social distancing (3); state and local municipalities issuing stay at home orders (4, 5); indefinite closure of many non-essential businesses such as restaurants, shopping malls, gyms, and fitness centers (5); and many businesses and organizations shifting from in-office work to remote (from home) work (6, 7). Coverage of the pandemic dominated news and social media platforms, further adding to the distress experienced by many Americans (8). As with other countries, these events disrupted the daily activities of many Americans, and anecdotally, their health behaviors, including physical activity (PA).

PA is an established behavior for the promotion of overall health and wellness. Performing regular PA is inversely associated with the development of cardiometabolic diseases (i.e., cardiovascular disease, type 2 diabetes, obesity) (9) and certain cancers (i.e., colorectal, breast, and prostate) (10), enhances mood and psychological well-being (11, 12), and has a strong, inverse dose-response with cardiovascular and all-cause mortality (9). Although PA provides health benefits at any age, engaging in regular PA becomes even more important as individuals transition from midlife to older age. PA during mid-life and older age further reduces risk for developing cardiometabolic disease, stroke, and acute cardiovascular events (i.e., myocardial infarction) (9), mitigates functional limitations associated with increased age and reduces risk for falls (13, 14), attenuates declines in bone density (15), and delays cognitive decline and the onset of Alzheimer's disease and related dementias (16). Given the myriad health benefits of regular PA, it should come as no surprise that PA has also been recommended as a strategy to promote immune function to reduce risk for severe COVID-19 outcomes, enhance COVID-19 vaccine efficacy, and assist with management of pandemic related stress, anxiety, and depression (17, 18).

Recently published data from countries outside of the US (19–40) suggest that the early stages of the SARS-CoV-2 pandemic were associated with decreased PA among adults. However, at present, no published studies have reported the impact of the early stages of the pandemic on the PA levels of US adults. Likewise, only a few published studies (25, 30, 39) examining the impact of the pandemic on PA behaviors have included a substantial number of middle-aged and older adults, limiting knowledge about how the pandemic affected PA patterns among this high-risk group for developing severe COVID-19 outcomes (i.e., hospitalization and death) (41, 42).

The purpose of this study was to examine the impact of the SARS-CoV-2 pandemic on the PA pattern of US adults aged 50 years and older. The primary aim was to longitudinally examine patterns of PA change from before the SARS-CoV-2 pandemic (retrospectively reported) and at two time points during the early months of the pandemic (i.e., between May-June 2020 and between June-July 2020). We hypothesized that participants would report lower PA levels during the pandemic when compared to pre-pandemic levels. A secondary aim was to identify predictors of PA change, including sociodemographic characteristics (age, gender, race, ethnicity, relationship status, educational status, employment status, income), perceived COVID-19 threat, self-reported general health, and presence of chronic disease. Given the novelty of the virus and our emerging understanding of the sequelae of COVID-19, these analyses were exploratory with two exceptions. Those reporting better general health and free of chronic disease were hypothesized to exhibit greater maintenance of PA during the pandemic, as compared to those of poorer health or with chronic disease (43–45). Other hypotheses were less clear with respect to directionality. Those who perceived the virus and the disease as more threatening may have exercised less due to fears of exposure at gyms and other public spaces. Alternatively, they may have exercised more as an active coping strategy (e.g., to boost their immune system). Regarding socioeconomic factors, individuals of lower education and income status may not have had the option to work remotely from home. This either kept them active (e.g., if their job involved physical labor or being on their feet) or afforded less opportunity for leisure-time PA given other pandemic-related demands such as child care. Those working from home may have had more opportunity for exercise (bonus time gained from no commute), or exercised less given gym closures and other social distancing measures that kept their activities restricted. Regarding gender, there was suggestion early on in the media that the effects of COVID-19 were worse for men vs. women. Indeed, some evidence has borne this out, not greater incidence but greater case mortality, in particular among older men (46). This lay knowledge could have restricted activity among older males. Mechanisms underlying these hypothetical associations are likely multifaceted, spanning the biological to the behavioral (47). Our intent here was not to elucidate such mechanisms, but to examine change over time in PA during the pandemic and to identify potential risk and protective factors.

## Methods

### Study Design, Population, and Recruitment

Data were collected as part of the Aging in the Time of COVID-19 Study, a longitudinal, web-based, multi-wave North American survey study examining the influence of the SARS-CoV-2 pandemic on the health and well-being of middle-aged and older adults. Data included in this report were collected during the first three study waves. Wave 1 was conducted between April 13 and May 15, 2020, a time characterized by the onset of physical and social distancing guidelines, initial closure of many non-essential businesses, and employers transitioning employees to remote work. Participants completed Waves 2 and 3 ~30 and 60 days, respectively, after their Wave 1 assessment (i.e., Wave 2 was conducted between May 11 and June 7, 2020 and Wave 3 between June 1 and July 1, 2020). Participants were recruited *via* advertisements on email listservs, university forums, and social media platforms (i.e., Twitter and Facebook). Individuals were eligible for study participation if they were aged 50 years or older, English speaking/reading, and resided in North America. Data reported in this article are restricted to participants residing in the US, which included representation from 46 of 50 states (i.e., no participants reported residing in Oklahoma, Vermont, West Virginia, or Wyoming), as the purpose was to examine the impact of the pandemic on the PA patterns of US adults aged 50 years and older. Electronic Supplementary Material 1 includes the frequency of participants from each US state. REDCap electronic data capture tools hosted by Arizona State University (48, 49) were used to administer the survey and collect all study data. As compensation for participation, participants had the option to provide their contact information to enter a raffle for a $25 gift card after completing each wave. Informed consent was obtained from all participants and all study procedures were approved by the Institutional Review Board of Arizona State University.

### Measures

#### Demographics

Demographic characteristics, assessed at Wave 1 using items adapted from the 2017 Behavioral Risk Factor Surveillance System (BRFSS) Questionnaire (50), included age, gender, race, ethnicity, relationship status, education, income, and employment status.

#### Physical Activity

Physical activity (PA) was assessed at Waves 2 and 3 using the International Physical Activity Questionnaire-Short Form (IPAQ-SF) (26). The IPAQ-SF provides an estimate of weekly energy expenditure in metabolic equivalent (MET)-minutes/week based on time spent walking (at work and at home, to travel from place to place, and for recreation, sport, exercise, or leisure), in moderate-intensity activities (carrying light loads, bicycling at a regular pace, or doubles tennis), and in vigorous-intensity activities (heavy lifting, digging, aerobics, or fast bicycling). The sum of these three intensities was also calculated to provide an estimate of total PA. At Wave 2, participants completed IPAQ twice, once in a retrospective manner with reference to PA during a normal 7-day period before social and physical distancing was recommended to prevent transmission of SARS-CoV-2 (henceforth termed pre-pandemic PA), and again with reference to the past 7 days. At Wave 3, participants completed the IPAQ-SF once, in reference to the past 7 days. This approach provided an estimate of PA at three referents: (1) a typical 7-day period before the pandemic (retrospectively assessed at the Wave 2 assessment), (2) at Wave 2 with regard to the past 7 days, and (3) at Wave 3 (June 1–July 1, 2020) with regard to the past 7 days. All of these data were scored according to 2005 IPAQ protocols (51).

#### Perceived COVID-19 Threat

Perceived COVID-19 threat was assessed at Wave 2 using a 7-item questionnaire developed by Conway et al. (52). Using a Likert-type scale ranging from 1 to 7 (1 = not true of me at all; 7= very true of me), participants responded to various statements assessing perceived threat of contracting and transmitting coronavirus (i.e., SARS-CoV-2). Example items included, “Thinking about the coronavirus (COVID-19) makes me feel threatened” and “I am worried that I or people I love will get sick from the coronavirus (COVID-19).” The questionnaire was scored by calculating the mean of all seven items, with higher scores indicating greater perceived threat. The questionnaire demonstrated adequate internal consistency (Cronbach alpha = 0.75).

#### Self-Rated General Health

Self-rated general health was assessed at Wave 1 using The World Health Organization's single-item health questionnaire (53). This item asked participants, “In general, how would you rate your health today?” Response options included: 1 = very good, 2 = good, 3 = moderate, 4 = bad, and 5 = very bad. Reponses were reverse coded for all outcome analyses, with higher scores indicating better health (i.e., 1 = very bad health; 5 = very good health).

#### Chronic Disease Conditions

The presence or absence of chronic disease was assessed at Wave 1 using items adapted from the 2017 BRFSS (50). Nine conditions were assessed: (a) angina or coronary heart disease; (b) stroke; (c) chronic obstructive pulmonary disease; emphysema or chronic bronchitis; (d) arthritis; (e) kidney disease; (f) diabetes; (g) osteoporosis; (h) Alzheimer's disease or related dementias; and (i) cancer (any type excluding skin cancer; skin cancer was assessed as a separate item but did not differentiate between basal cell carcinoma and melanoma; because basal cell is generally not considered a chronic disease, this item was excluded from the aggregate). The sum of these conditions served as a continuous variable in outcomes analyses. The theoretical range for this summary score was 0 to 9.

### Statistical Analysis

#### Data Analysis

We first examined basic descriptive statistics and frequencies to identify if implausible values were present, determine the extent of incomplete data, and screen for possible violations of assumptions. As expected given the longitudinal design, some outcome variables exhibited non-normality, and data for some variables were incomplete. We treated each of these issues as described below. We also examined values of the variance inflation factor to assess multicollinearity and measures of influence (e.g., Cook's distance) to identify potential influential observations.

To assess the degree to which mean PA changed across study periods, we conducted a multivariate repeated measures analysis separately for each PA intensity (i.e., walking, moderate, vigorous) and total PA with time referent (i.e., Wave 2 pre-pandemic, Wave 2 past 7 days, and Wave 3 past 7 days) as the repeated measures factor. Parameters were estimated with maximum likelihood estimation and robust standard errors using Mplus software (Version 8.5) (54), which is robust to violations of normality and provides for optimal parameter estimates when response data are incomplete (55). Although this procedure provides state-of-the-art missing data treatment (56), we included missing data correlates, or auxiliary variables, to further improve parameter estimation and enhance statistical power (56–58). Given that the most useful auxiliary variables are those that have correlations with the incomplete analysis variables that exceed a magnitude of 0.40 (59), we used the saturated correlates model (57) to include such auxiliary variables, which were always the other PA timepoint referents. We used statistical tests available in Mplus, the multivariate Wald test and *z* test, to assess the mean change across periods as well as pairwise comparisons between specific time referents. We also computed Cohen's *d* type effect size measures by dividing a given pairwise mean difference by the standard deviation of the outcome at the earlier period.

**Figure 1:**
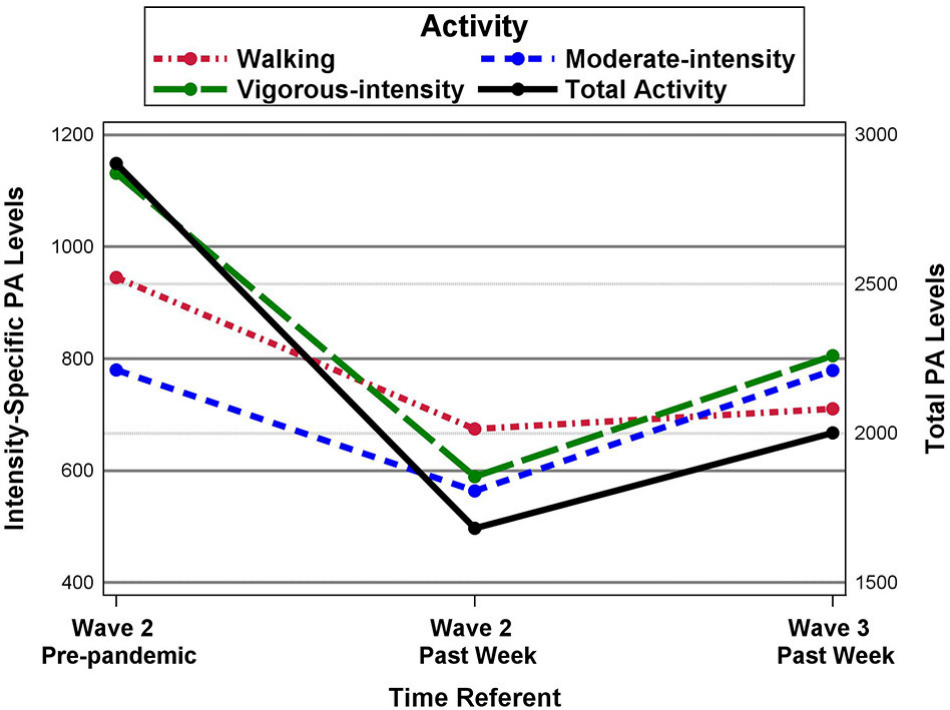
Longitudinal changes in MET-minutes/week of total and intensity-specific physical activity.

To determine which variables were predictive of change in total PA, we computed three sets of difference scores between each time referent (pre-pandemic to Wave 2, Wave 2 to Wave 3, pre-pandemic to Wave 3) and estimated a regression model for each of the three difference scores. The predictors in each model were the same and included demographic characteristics, total PA at the pre-pandemic time referent, perceived COVID-19 threat, self-rated general health, and number of chronic diseases. Demographic characteristics treated as predictors were age (continuous), gender (1 = female, 0 = male), race (1 = white, 0 = other), ethnicity (1 = Hispanic, 0 = non-Hispanic), relationship status [1 = partnered (married or member of an unmarried couple), 0 = other], employment status [1 = employed (full- or part-time or self-employed), 0 = not employed (unemployed, homemaker, student, retired, unable to work)], educational status (represented by two-dummy coded predictors, with the no college degree group serving as the reference group, as the high school group was removed from the regression analysis due to excessive multicollinearity, as described below), and total household income (represented by two dummy-coded predictors with income < $50,000 serving as the reference group). As in the previous analysis, maximum likelihood estimation with robust standard errors was used to estimate parameters and treat incomplete data (54), which included incomplete outcome and predictor variables. As a result, neither in the previous nor in this set of analyses were any cases omitted due to incomplete data. In addition, because Mplus does not allow variables to be included in the analysis if their variance exceeds one million, we rescaled each PA outcome by dividing the raw scores by 100 for analyses. This transformation does not affect the values of statistical tests, their corresponding *p*-values, or the estimates of effect size (i.e., *R*^2^, standardized mean differences or standardized regression coefficients). Descriptive results of PA outcomes were subsequently rescaled (i.e., multiplied by 100) for the reporting of study outcomes. Alpha was set at 0.05 for all tests. SAS software (version 9.4) was used to create Figure 1.

#### Power and Sample Size

With our large sample size (*N* = 589), analyses had ample statistical power to detect all but trivial effects. For the repeated measures analyses, power analyses conducted *via* PASS statistical software (60) indicated that power exceeded 0.99 to detect a difference in means equal to 0.2 standard deviations (often regarded as a small effect) and exceeded 0.82 for a mean difference of 0.12 standard deviations, given use of a two-tailed test and an alpha of 0.05. With the same alpha level, a sample size of 573 for the regression analyses provides power that exceeded 0.95 to detect the effect of a given predictor, assuming the predictor accounts for at least 2% of the unique variation in the outcome (i.e., Δ*R*^2^ = 0.02), given that the remaining 13 predictors account for 13% of the total outcome variance.

## Results

### Participant Characteristics

Table 1 presents descriptive data for participant demographic characteristics and other predictors. Briefly, participants (*N* = 589) had a mean age of 63 ± 7.39 years and most were female (88%), non-Hispanic (96%), White (96%), and married (63%). Two-thirds had earned a bachelor's degree or higher (77%) and less than half (45%) reported being employed (i.e., full-time, part-time, or self-employed). Household income varied, with almost a quarter of participants reporting an income of < $50,000 (24%); the remaining participants reported incomes of $50,000 to $99,999 (36%) or >$100,000 (37%). The majority of participants (85%) reported being in good-to-very good health based on the general health question (*M* = 4.15 out of 5), and most (59%) did not have a chronic disease condition. Respondents also reported a relatively high level of perceived COVID-19 threat (*M* = 4.54 out of 5.0).

**Table 1:** Participant (*N* = 589) demographic characteristics and descriptive outcomes for perceived COVID-19 threat, health status, and chronic disease conditions.

**Variable**	***M***	**SD**	***n***	**%**
Age,	63.2	7.39		
**Gender**
Male			69	11.7
Female			520	88.3
**Ethnicity**
Hispanic/Latino			20	3.4
Non-Hispanic/Latino			565	95.9
Did not report			4	0.7
**Race**
Black			1	0.2
Pacific Islander			2	0.3
White			566	96.1
Other			15	2.6
Did not report			5	0.8
**Education**
High school grad or GED			16	2.7
Some college or technical school			109	18.5
Bachelor's degree			171	29.0
Graduate school			282	47.9
Did not report			11	1.9
**Income**
Less than $50,000			140	23.8
$50,000-$99,999			213	36.2
$100,000+			217	36.8
Did not report			19	3.2
**Employment Status**
Employed			264	44.8
Unemployed/unable to work			55	9.5
Homemaker			22	3.7
Student			4	0.7
Retired			239	40.6
Did not report			5	0.8
**Relationship status**
Married			373	63.3
Divorced			96	16.3
Widowed			41	7
Separated			7	1.2
Never married			41	7.0
Unmarried couple			30	5.1
Did not report			1	0.2
Perceived COVID-threat^a^	4.54	1.10		
Single-item health rating^b^	4.15	0.75		
Frequency of chronic	0.57	0.81		
disease conditions^c^
0			345	58.6
1			181	30.7
2			44	7.5
3			14	2.4
4			3	0.5
5			2	0.3
**Frequency of specific chronic disease conditions**
Coronary Heart Disease			30	5.1
Stroke			10	1.7
Chronic Obstructive Pulmonary Disease (COPD)			26	4.4
Arthritis			47	8.0
Kidney Disease			20	3.4
Diabetes			69	11.7
Osteoporosis			56	9.5
Alzheimer's or Related Dementias			1	0.2
Cancer (excluding skin cancer)			74	12.6

a *Available range for perceived COVID-threat score was 1–5*.

b *Available range for single-item health rating score was 1–5*.

c *Range for number of chronic disease conditions reported was 0–5*.

### Longitudinal Changes in Physical Activity

Table 2 shows changes in PA by assessment period. Participants reported performing a total of 2,904 MET-minutes/week of pre-pandemic PA, with 945 MET-minutes/week being performed in walking activities, 780 MET-minutes/week in moderate-intensity activities, and 1,131 MET-minutes/week in vigorous-intensity activities. Wald tests examining PA changes across assessment periods indicated mean PA differences were present for each PA outcome (i.e., walking, moderate-intensity, vigorous-intensity, and total PA). *Z-*tests showed PA declined from Wave 2 pre-pandemic to Wave 2 past 7 days, with Cohen's *d* indicating similar mean declines for all three PA intensity levels (*d* = −0.28 for walking, *d* = −0.21 for moderate-intensity, and *d* = −0.26 for vigorous-intensity PA) and a larger mean decline in total PA (i.e., *d* = −0.45). From Wave 2 past 7 days to Wave 3, moderate-intensity and vigorous-intensity PA significantly increased (i.e., increases of 215 and 216 MET-minutes/week, with *d* values = 0.29 and 0.17, respectively), with moderate-intensity PA returning to pre-pandemic levels (i.e., ~780 MET-minutes/week). In contrast, at Wave 3, walking-intensity PA, vigorous-intensity PA, and total PA remained significantly below pre-pandemic levels. Figure 1 displays the PA means across the three time referents for total and intensity-specific PA outcomes.

**Table 2:** Mean MET-min/week of physical activity by intensity-level and referent time period.

	**Time referent**			**Omnibus**	**Comparisons**					
**Activity**	**Wave 2 pre-pandemic**	**Wave 2 past week**	**Wave 3 past week**	**Wald**	**Wave 2 vs. pre-pandemic**		**Wave 3 vs. wave 2**		**Wave 3 vs. pre-pandemic**	
**Intensity**	**M (SD)**	**M (SD)**	**M (SD)**	**Test**	**MD (SE)**	***d***	**MD (SE)**	***d***	**MD (SE)**	***d***
Walking	945 (971)	675 (899)	711 (902)	31.1^***^	−271^***^ (49)	−0.28	36 (53)	0.04	−235^***^ (65)	−0.24
Moderate	780 (1,034)	564 (739)	779 (1,043)	29.6^***^	−216^***^ (49)	−0.21	215^***^ (57)	0.29	−1 (69)	−0.01
Vigorous	1,131 (2,107)	589 (1,306)	805 (1,690)	40.4^***^	−542^***^ (88)	−0.26	216^*^ (93)	0.17	−326^**^ (121)	−0.16
Total	2,904 (2,691)	1,682 (2,044)	2,001 (2,491)	125.2^***^	−1222^***^ (111)	−0.45	319 (135)	0.16	−904^***^ (169)	−0.34

*N = 589. MD = mean difference*.

* *P < 0.05*.

** *P < 0.01*.

*** *P < 0.001*.

### Predictors of Physical Activity Change

Regression analyses of time specific changes in total PA indicated substantial multicollinearity for the predictor educational status, with the associated dummy-coded variables having variance inflation factors ranging from 6.2 to 10. Dropping those with only a high school education (*n* = 16) remedied the problem with no variance inflation factor >2.6 for the remaining cases. The regression results were similar for the change in total PA scores during specific time points. Specifically, as shown in Table 3, Wave 2 pre-pandemic PA was predictive of the change from one from time point to the next, such that participants with higher levels of pre-pandemic PA experienced a greater decline in activity from Wave 2 pre-pandemic to Wave 2 past 7 days (β = −0.74, *p* < 0.001) and Wave 3 (β = −0.71, *p* < 0.001), along with a smaller increase in activity from Wave 2 past 7 days to Wave 3 (β = −0.41, *p* < 0.001). In addition, general health was associated with changes in activity between each period. Participants indicating better general health reported smaller declines in total PA from the pre-pandemic period to Wave 2 past 7 days (β = 0.14, *p* < 0.001) and Wave 3 (β = 0.17, *p* < 0.01), and a greater increase or rebound in PA from Wave 2 past 7 days to Wave 3 (β = 0.14, *p* < 0.05). No other demographic or predictor variables were associated with PA changes. Although pre-pandemic PA levels and general health were the only two predictors significantly associated with PA change, the predictors, as a set, accounted for over 50% of the variation in the change in total activity from the pre-pandemic time referent to Wave 2 past 7 days and from Wave 2 pre-pandemic to Wave 3, as well as 19% of the variation in the total PA change from Wave 2 to Wave 3.

**Table 3:** Regression results for total MET-min/week of activity change between specific periods.

	**Wave 2 vs. pre-pandemic**			**Wave 3 vs. wave 2**			**Wave 3 vs. pre-pandemic**		
**Predictor**	**B^**a**^**	***SE***	***β^*b*^***	***B***	***SE***	**β**	***B***	***SE***	**β**
Age	−7.5	12.0	−0.02	24.5	19.1	0.07	28.3	21.3	0.06
Female sex	−339.5	243.4	−0.13	−267.7	374.1	−0.11	−434.3	425.8	−0.13
White race	−417.6	245.6	−0.16	16.2	687.2	0.01	−223.4	794.2	−0.06
Hispanic Ethnicity	93.8	438.2	0.04	1040.3	834.6	0.42	997.9	844.9	0.29
Partnered relationship status	41.7	194.4	0.02	257.6	320.6	0.11	395.9	379.9	0.11
Employed	122.0	175.8	0.05	432.2	276.6	0.18	455.0	301.0	0.13
**Education**	
Bachelors vs. no college degree	149.1	210.8	0.06	429.0	420.2	0.17	529.4	470.5	0.15
Graduate school vs. no college degree	195.7	207.8	0.08	−87.9	392.8	−0.04	−54.7	452.3	−0.02
**Income**									
50 K to 100 k vs. <50 K	−183.6	243.9	−0.07	171.0	435.3	0.07	131.5	490.5	0.04
100 K+ vs. <50K	−316.7	253.6	−0.12	−150.1	447.8	−0.06	−427.5	505.9	−0.12
Pre-pandemic activity	–71.3^***^	3.0	–**0.74**	–50.4^***^	8.1	–**0.41**	–91.1^***^	5.2	–**0.71**
Covid-19 threat	−17.6	69.3	−0.01	177.2	121.2	0.08	158.5	128.2	0.05
Single-item general health	477.7^***^	101.2	**0.14**	470.7^*^	205.9	**0.14**	782.1^**^	226.0	**0.17**
Number of chronic disease conditions	−53.6	90.2	−0.02	200.5	175.1	0.07	142.5	191.6	0.03
Intercept	220.4	1015.1	0.08	−3536.6	2225.5	−1.47	−3916.2	2284.6	−1.13
*R*^2^	0.54^***^			0.19^***^			0.52^***^		

*N = 573. Female is coded as female = 1, male = 0. White is coded as white = 1, other = 0. Hispanic is coded as 1 = Hispanic origin, 0 = otherwise. Couple is coded as 1 = married or unmarried couple, 0 = otherwise. Employed is coded as 1 = employed, 0 is unemployed*.

a *B is an unstandardized regression coefficient*.

b β is a standardized regression coefficient. For the dummy-coded predictors, β= B/sdy. For the numeric predictors, β= (B*sdx)/sdy.

* *p < 0.05*.

** *p < 0.01*.

*** *p < 0.001. Bold values indicate statistically significant outcomes*.

## Discussion

To our knowledge, this is the first published study to report the impact of the SARS-CoV-2 pandemic on the PA patterns of US adults. Results showed that among our sample of middle-aged and older adults, PA significantly declined during the early stages of the pandemic (i.e., May through June 2020). This finding mirrors results of numerous international (20, 61) and country-specific studies emerging from Europe (24, 25, 29, 34, 37, 43, 62), Asia (38), and Australia (63), including those focused exclusively on middle-aged and older adults (30, 62). It also bolsters the notion that the pandemic has adversely affected critical lifestyle behaviors, in this case PA, which is known to be protective for physical and mental health and disease prevention (9, 10, 12, 16, 64). Moreover, given regular PA is an established behavior to prevent and minimize weight gain (65), our findings may lend some support for a hypothesized new pandemic on the horizon: “covibesity.” Khan and Moverly Smith postulated in a recent letter to the editor of *Obesity Medicine* (66) that decreased PA and increased energy intake (resulting from increased food shopping, food take away, alcohol sales, and psychological distress) during the SARS-CoV-2 pandemic is leading to rapid weight gain, a term they coined as “covibesity.” Although empirical data have yet to support the realization of this impending “pandemic,” should it bear out, our data may provide important information on at least one of its determinants, decreased PA.

While significant decreases in PA from “pre-pandemic” levels to Wave 2 are cause for alarm, further decreases in PA were not observed at Wave 3. Instead, slight increases were made for moderate- and vigorous-intensity PA; however, overall PA levels remained significantly below “pre-pandemic” levels. Speculatively, this trend may suggest that participants were gradually increasing their PA over time as more information became available on how the novel coronavirus is transmitted (i.e., predominantly airborne) and effective mitigation strategies (i.e., social distancing, wearing a mask, being outdoors when gathering with individuals who reside outside of one's household). However, research is needed to tease out these mechanisms or to explore cognitions behind health behaviors such as PA during the pandemic. Independent of the pandemic, longitudinal studies show PA levels of most middle-aged and older adults gradually decline overtime (67). Of concern is that the pandemic will accelerate longitudinal declines in PA and the possibility that the majority of middle-aged and older adults will never again achieve their pre-pandemic PA levels. Should these scenarios occur, the US may observe subsequent increases in morbidity and mortality among older adults from conditions directly associated with low PA levels in the coming years, including cardiometabolic diseases (i.e., cardiovascular disease, type 2 diabetes, stroke) and Alzheimer's disease and related dementias.

Another key finding was that higher levels of pre-pandemic PA were associated with greater decreases in PA during the pandemic. A similar outcome was recently reported by authors of a large United Kingdom study (n = 5,395; *M* age 41 years) examining objectively-measured PA collected from January 22 to June 17, 2020 through a commercially available physical activity smartphone application (68). We hypothesize that the highly active “pre-pandemic” adults in our sample performed structured leisure-time activities (i.e., tennis, aerobics classes, scheduled walking with friends/family), as opposed to only getting their PA through activities of daily living. Given that the majority of fitness and community centers in the US were closed during the early stages of the pandemic to reduce transmission of SARS-CoV-2 and many Americans transitioned from in-office work to remote work, possibly limiting some participants' ability to utilize fitness centers or exercise equipment available at their place of employment, these individuals were likely unable to maintain their usual leisure-time PA routines, resulting in a marked decrease in PA. A recently published qualitative study with older adults residing in France (30) supports this assumption, as results of this study showed PA levels among older adults were reduced during the pandemic due to the cancellation of group-based exercise classes and/or participants not wanting to participate in group sessions for fear of contracting COVID-19. However, future research on this topic is needed before definitive conclusions can be drawn.

Self-reported general health also emerged as a significant predictor of PA change over time. Better general health was associated with more attenuated declines in PA during the pandemic. A reason for this may be that individuals who self-report being in good or very good health value the health benefits of PA and identified ways to be active despite barriers imposed by the pandemic (i.e., home-based PA and/or socially distanced outdoor PA). It might also simply be easier for these individuals to engage in PA due either to better physical function or to engrained healthy habits. The mechanism of course is unclear but a strength of our study is the fact that self-reported health preceded the measurement of PA in time.

An unexpected finding was that frequency of chronic disease conditions was not associated with PA change. This outcome contradicts a recent study (43) demonstrating that a higher number of chronic disease conditions was associated with greater decreases in PA during the SARS-CoV-2 pandemic. In the absence of the pandemic, studies have consistently shown an inverse relationship between the number of chronic disease conditions and longitudinal changes in PA (i.e., higher number of chronic disease conditions, greater decrease in PA over time) (44, 45). A reason for our null finding could be related to most of our sample reporting no (59%) or only one (31%) chronic disease condition, limiting power to examine this relationship. Likewise, although participants reported relatively high levels of COVID-19 threat, this variable was not associated with changes in PA. This may be due to a ceiling effect, allowing for limited variance to examine this predictor.

Limitations of the study include the use of self-reported PA measures and having participants retrospectively assess PA prior to the pandemic. Self-reported PA measures are associated with recall bias and generally reflect higher levels than objectively measured PA. A prospective design was simply not possible in this case, as this survey was created in response to the pandemic. Additionally, data are limited to only 3 time referents during the early stages of the pandemic (i.e., prior to the pandemic and two time points during the early stages of the pandemic). We acknowledge that it would have been beneficial for the research team to continue to follow participants during the pandemic to provide more detailed information on the longitudinal PA patterns of our sample. However, this was beyond the scope of the project as initially conceived. Another limitation is that the study design does not allow us to tease out the role of seasonality on PA outcomes. Participants were from diverse regions of the US (see Supplementary Table 1) and the unique role of seasonality on PA levels likely differed based on geographic location and assessment period. Given that the study did not include an objective assessment of weather patterns or subjective items regarding any impacts of weather on PA, we are unable to determine how seasonality influenced PA outcomes. A fourth limitation is that our sample comprised relatively highly educated, middle-to-upper class White women, limiting generalizations to men, women of different races, and individuals of lower socioeconomic status. Future research is warranted to explore the impact of the SARS-CoV-2 pandemic on PA patterns in a more diverse US sample. Lastly, because of the numerous online recruitment strategies employed (i.e., email listservs, university forums, and social media platforms (i.e., Twitter and Facebook) we are unable to determine the reach of our recruitment methods to calculate a recruitment rate.

Despite these limitations, the study has several strengths. To our knowledge, this is the first study to report how the SARS-CoV-2 pandemic has affected the PA patterns of US adults. Similarly, our study is one of few studies to describe a large sample of middle-aged and older adults, regardless of country of origin. Findings provide important insight into how the pandemic affected PA among this unique population of middle-aged and older adults, who are not only at greater risk for severe COVID-19 outcomes, but also arguably, at the greatest need for regular PA engagement. Another strength of the study was that our design allowed for examination of PA levels at multiple time points during the pandemic, that is, ability to describe trajectories of change across this historical period. A final strength was our relatively large sample from diverse areas of the US.

## Conclusions

Results suggest that the SARS-CoV-2 pandemic adversely affected the daily PA patterns of middle-aged and older US adults. Although programs that encourage and facilitate PA are always of importance, our data show that there is a critical need for researchers and public health professionals to identify effective strategies to promote safe and socially distanced PA until the risk of contracting COVID-19 subsides. Moreover, in the post-pandemic era, there will likely be an increased need for effective PA programming to increase PA among sedentary middle-aged and older adults. Such interventions will be imperative to ensure pandemic-related decreases in PA do not impact long-term health trajectories of middle-aged and older Americans.

## Data Availability Statement

The raw data supporting the conclusions of this article will be made available by the authors, without undue reservation.

## Ethics Statement

The studies involving human participants were reviewed and approved by Arizona State University Institutional Review Board. Participants provided informed consent at the beginning of the survey by agreeing to complete the survey.

## Author Contributions

RJ and SL were involved in conceptualizing the research questions examined in the manuscript, data analysis, and writing the manuscript. KP performed data analysis and assisted with writing of the manuscript. MG, MM, and AP are the primary investigators of the COVID in the Time of Aging study and participated in data acquisition and manuscript preparation. DC and WK participated in manuscript preparation. All authors contributed to the article and approved the submitted version.

## Conflict of Interest

The authors declare that the research was conducted in the absence of any commercial or financial relationships that could be construed as a potential conflict of interest.
