# Supplementary material for: Physical Activity Among Predominantly White Middle-Aged and Older US Adults During the SARS-CoV-2 Pandemic: Results From a National Longitudinal Survey
Source: Front Public Health. 2021 Apr 13;9:652197. doi: 10.3389/fpubh.2021.652197 (PMC8076643; doi:10.3389/fpubh.2021.652197)
Supplement: Supplementary file 1 [file Table_1.DOCX]

**Electronic Supplementary Material 1.**

*Number of participants residing in each U.S. state.*

| State | n | % |
| --- | --- | --- |
| Arizona | 134 | 22.8 |
| California | 80 | 13.6 |
| Michigan | 21 | 3.6 |
| Ohio | 21 | 3.6 |
| New Mexico | 19 | 3.2 |
| New York | 19 | 3.2 |
| Pennsylvania | 19 | 3.2 |
| Illinois | 18 | 3.1 |
| Texas | 16 | 2.7 |
| Florida | 15 | 2.5 |
| Oregon | 15 | 2.5 |
| Colorado | 14 | 2.4 |
| Missouri | 13 | 2.2 |
| Washington | 13 | 2.2 |
| Nevada | 12 | 2 |
| North Carolina | 11 | 1.9 |
| Virginia | 11 | 1.9 |
| Massachusetts | 10 | 1.7 |
| Wisconsin | 10 | 1.7 |
| Georgia | 9 | 1.5 |
| Louisiana | 9 | 1.5 |
| Minnesota | 7 | 1.2 |
| New Jersey | 7 | 1.2 |
| South Carolina | 7 | 1.2 |
| Alabama | 6 | 1 |
| Connecticut | 6 | 1 |
| Kansas | 6 | 1 |
| Maryland | 6 | 1 |
| Tennessee | 6 | 1 |
| Indiana | 5 | 0.8 |
| Maine | 5 | 0.8 |
| Mississippi | 5 | 0.8 |
| Delaware | 4 | 0.7 |
| Utah | 4 | 0.7 |
| Alaska | 3 | 0.5 |
| Arkansas | 3 | 0.5 |
| Idaho | 3 | 0.5 |
| Kentucky | 3 | 0.5 |
| Nebraska | 3 | 0.5 |
| Hawaii | 2 | 0.3 |
| Iowa | 2 | 0.3 |
| North Dakota | 2 | 0.3 |
| Rhode Island | 2 | 0.3 |
| Montana | 1 | 0.2 |
| New Hampshire | 1 | 0.2 |
| South Dakota | 1 | 0.2 |
| Oklahoma | 0 | 0 |
| Vermont | 0 | 0 |
| West Virginal | 0 | 0 |
| Wyoming | 0 | 0 |
| Total | 589 | 100.0 |
|  |  |  |
